# Gender-specific association of body composition with inflammatory and adipose-related markers in healthy elderly Europeans from the NU-AGE study

**DOI:** 10.1007/s00330-018-5973-2

**Published:** 2019-02-04

**Authors:** Aurelia Santoro, Giulia Guidarelli, Rita Ostan, Enrico Giampieri, Cristina Fabbri, Claudia Bertarelli, Claudio Nicoletti, Fawzi Kadi, Lisette C. P. G. M. de Groot, Edith Feskens, Agnes Berendsen, Anna Brzozowska, Olga Januszko, Katarzyna Kozlowska, Susan Fairweather-Tait, Amy Jennings, Nathalie Meunier, Elodie Caumon, Alessandro Napoli, Daniele Mercatelli, Giuseppe Battista, Miriam Capri, Claudio Franceschi, Alberto Bazzocchi

**Affiliations:** 1 Department of Experimental, Diagnostic and Specialty Medicine, Alma Mater Studiorum, University of Bologna, Via Massarenti 9, 40138 Bologna, Italy; 2 C.I.G. Interdepartmental Centre “L. Galvani”, Alma Mater Studiorum, University of Bologna, Bologna, Italy; 3 Department of Physics, Alma Mater Studiorum, University of Bologna, Bologna, Italy; 4 Department of Experimental and Clinical Medicine, Section of Anatomy, University of Florence, Florence, Italy; 5 Gut Health Institute Strategic Programme, Quadram Institute Bioscience, Norwich, UK; 6 School of Health and Medical Sciences, Örebro University, Örebro, Sweden; 7 Department of Human Nutrition and Health, Wageningen University, Wageningen, The Netherlands; 8 Department of Human Nutrition, WULS-SGGW, Warsaw, Poland; 9 Norwich Medical School, University of East Anglia, Norwich, UK; 10 CHU Clermont-Ferrand, CRNH Auvergne, 63000 Clermont-Ferrand, France; 11 Radiology Section, Department of Radiological, Oncological and Anatomopathological Sciences, Sapienza University of Rome, Rome, Italy; 12 Diagnostic and Interventional Radiology, IRCCS Istituto Ortopedico Rizzoli, Bologna, Italy; 13 Institute of Neurological Sciences (IRCCS), Bologna, Italy

**Keywords:** Aging, Body composition, DXA, Inflammation, Sarcopenia

## Abstract

**Objectives:**

The aim of this work was to examine the cross-sectional relationship between body composition (BC) markers for adipose and lean tissue and bone mass, and a wide range of specific inflammatory and adipose-related markers in healthy elderly Europeans.

**Methods:**

A whole-body dual-energy X-ray absorptiometry (DXA) scan was made in 1121 healthy (65–79 years) women and men from five European countries of the “New dietary strategies addressing the specific needs of elderly population for a healthy aging in Europe” project (NCT01754012) cohort to measure markers of adipose and lean tissue and bone mass. Pro-inflammatory (IL-6, IL-6Rα, TNF-α, TNF-R1, TNF-R2, pentraxin 3, CRP, alpha-1-acid glycoprotein, albumin) and anti-inflammatory (IL-10, TGF-β1) molecules as well as adipose-related markers such as leptin, adiponectin, ghrelin, and resistin were measured by magnetic bead-based multiplex-specific immunoassays and biochemical assays.

**Results:**

BC characteristics were different in elderly women and men, and more favorable BC markers were associated with a better adipose-related inflammatory profile, with the exception of skeletal muscle mass index. No correlation was found with the body composition markers and circulating levels of some standard pro- and anti-inflammatory markers like IL-6, pentraxin 3, IL-10, TGF-β1, TNF-α, IL-6Rα, glycoprotein 130, TNF-α-R1, and TNF-α-R2.

**Conclusions:**

The association between BC and inflammatory and adipose-related biomarkers is crucial in decoding aging and pathophysiological processes, such as sarcopenia. DXA can help in understanding how the measurement of fat and muscle is important, making the way from research to clinical practice.

**Key Points:**

*• Body composition markers concordantly associated positively or negatively with adipose-related and inflammatory markers, with the exception of skeletal muscle mass index.*

*• No correlation was found with the body composition markers and circulating levels of some standard pro- and anti-inflammatory markers like IL-6, pentraxin 3, IL-10, TGF-β1, TNF-α, IL-6Rα, gp130, TNF-α-R1, and TNF-α-R2.*

*• Skeletal muscle mass index (SMI) shows a good correlation with inflammatory profile in age-related sarcopenia.*

**Electronic supplementary material:**

The online version of this article (10.1007/s00330-018-5973-2) contains supplementary material, which is available to authorized users.

## Introduction

The assessment of body composition (BC) is essential for the characterization of metabolic status [1]. The changes in BC that occur with aging are mainly related to three distinct processes: (i) a progressive decrease in lean mass (LM) and an increase in fat mass (FM) potentially leading to sarcopenia and sarcopenic obesity [2]; (ii) a redistribution of FM, central and visceral [3]; and (iii) a reduction in body height and bone mineral density (BMD) [4, 5]. Excessive body fat accumulation is an established risk factor for a multitude of chronic conditions, including insulin resistance and type 2 diabetes, cardiovascular disease (CVD), and certain cancers, that are among the major causes of deaths in US and European population [6, 7]. Metabolic diseases are characterized by an inflammatory status called metaflammation, a particular case of chronic inflammation driven by nutrient excess/overnutrition [8]. Recently, it has been hypothesized that metaflammation may precede/contribute to inflammaging, i.e., the chronic, low-grade, systemic, inflammatory state that characterizes aging [9, 10], and that metabolic age-related dysfunctions and diseases can be considered manifestations of aging acceleration [11]. Levels of major circulating pro-inflammatory cytokines, e.g., the tumor necrosis factor alpha (TNF-α), interleukin (IL)-1 family, IL-6, and IL-8, are increased in both conditions [12]. Interestingly, inflammaging does not simply reflect an increase of pro-inflammatory markers but an overall activation of inflammatory systems that probably also promotes a concomitant rise in the levels of anti-inflammatory mediators [13, 14]. As adipose tissue expands and muscle and bone tissue decrease during aging, there is an increase in pro-inflammatory and a reduction in anti-inflammatory adipokines, chemokines, and cytokines which contributes to local and systemic inflammation and disturbances in glucose homeostasis [15]. However, studies on the relationship between composition and regional distribution of adipose and lean tissue and bone mass and the relative inflammatory profile in healthy elderly subjects are almost completely missing. A widely used technique for the assessment of human BC [16] is represented by dual-energy X-ray absorptiometry (DXA) [1, 17]. In the current study, we aimed to evaluate associations of several inflammatory and adipose-related hormones with adipose, lean tissue, and bone mineral content measured by DXA in a representative sample of European 65+-year-old healthy adults participating in the “New dietary strategies addressing the specific needs of elderly population for a healthy aging in Europe” (NU-AGE) study. Such relationships are of interest in clinical practice to investigate the role of inflammation and regional body composition markers in aging, sarcopenia, and obesity-related diseases.

## Materials and methods

### Study design and participants

NU-AGE (http://www.nu-age.eu/) was a 1-year, multicenter, randomized, single-blind, controlled trial (registered with clinicaltrials.gov, NCT01754012) with two parallel groups (i.e., dietary intervention and control). The recruitment was carried out in five European centers in France, Italy, the Netherlands, Poland, and the United Kingdom (UK). The recruitment of participants has been described in detail previously [18–20]. Briefly, 2668 volunteers from the community aged 65–79 years, free of major overt chronic diseases, living independently, and free of dementia, were recruited to participate in the baseline assessment. Of the 2668 participants, 1512 were screened for inclusion and 1296 were eligible to participate in the NU-AGE trial. In this study, we included 1121 participants who completed the baseline DXA assessment in the five recruiting centers (France (*N* = 184; 16.4%), Italy (*N* = 236; 21%), the Netherlands (*N* = 233; 20.7%), Poland (*N* = 222; 19.8%), and UK (*N* = 246; 21.9%)).

### Assessment of body composition

A whole-body DXA scan was performed to measure total and regional body composition using the fan-beam densitometers described in the supplementary methods section.

Regions of interest were defined by the analytical software including six different corporeal districts: total body, trunk, upper limbs, lower limbs, android region (a portion of the abdomen included between the line joining the two superior iliac crests and extended cranially up to the 20% of the distance between this line and the chin), and gynoid region (a portion of legs from the femoral great trochanter, directed caudally up to a distance double of the android region). Android and gynoid regions were not defined by the densitometer used in the UK. For each region, DXA scanned the weight (in g) of total mass, FM, non-bone LM, and bone mineral content (BMC). The relationship between parameters derived from the different DXA machines was investigated using specific reliable indexes. In particular, total body FM/LM (a), fat mass index (FMI, whole-body fat mass/heigth^2^) (b), lean mass index (LMI, whole-body lean mass/heigth^2^) (c), android/gynoid FM (d), android FM/LM (e), appendicular lean mass index (ALMI, lean mass from arms plus legs/height^2^) (f), and skeletal muscle mass index (SMI, lean mass from arms plus legs/weight) (g) were considered as the pivotal markers of body composition, in terms of general mass balance (a, b, c), central/peripheral distribution of FM (d), central abdominal distribution (e), and low muscle mass (f, g), respectively. Moreover, BMD and T-score were also considered as markers of bone health [21].

In order to identify specific body composition profiles among the participants a cluster analysis was performed separately within women and men using the following ten BC markers: FM, FMI, LM, LMI, ALMI, FM/LM, SMI, T-score, BMC, and BMD in combination with BMI; the results of this analysis are described in [21]. Briefly, five clusters were identified for women (normal weight (NW), BMI = 21.39; overweight A (OWA), BMI = 25.09; overweight B (OWB), BMI = 26.62; low obesity A (LOA), BMI = 31.48; and low obesity B (LOB), BMI = 31.92) and six for men (NW, BMI = 23.98; OWA, BMI = 25.69; OWB, BMI = 26.27; LOA, BMI = 30.06; LOB, BMI = 30.42; and moderate obesity (MO), BMI = 36.6). These are able to discriminate groups of subjects with significantly different body composition markers when the BMI is very similar [22] (Supplementary Table 1).

### Statistical methods

According to the Shapiro–Wilk test for normality (*p* < .01), we decided to use non-parametric statistical tests. R project (version 3.3.3 for Windows) was used for the analysis, and results are reported as mean and standard deviation (± SD). Data were analyzed by Mann–Whitney and Kruskal–Wallis tests to determinate differences between men and women and between clusters [21]. We also used pairwise comparisons to test differences between all pairs of clusters. A type I error of .05 (*p* value) in two-tailed tests was considered significant. To assess a possible linear association between the body composition variables and markers of inflammation, we used the Pearson product-moment correlation, after a natural log-transformation (ln) for BC variables and a log-odds transformation for markers of inflammation. Due to multiple testing of the variables, the Benjamini–Hochberg correction was applied and both *p* value and *q* value are reported in “Results.”

## Results

### Participant characteristics

One thousand one hundred twenty-one subjects, 620 women (55%) and 501 men (45%), from the NU-AGE cohort were included in this study. Almost all the anthropometric, metabolic measures and the body composition markers considered were significantly different between men and women (Table 1), and for this reason, all the analyses were stratified by sex. Men had higher height, weight, waist circumference, waist-to-hip ratio, calorie intake, and glucose and hemoglobin levels than women (*p* < .05 for all). Women had significantly higher fat mass markers than men in terms of FM, FMI, FM/LM, and android FM/LM but lower android/gynoid FM. Conversely, men had significantly higher lean mass markers than women in terms of LM, ALMI, LMI, and SMI, and higher BMC and BMD than women. Higher levels of ghrelin, leptin, adiponectin, resistin, and alpha-1-acid glycoprotein (AGP) were found in women, but there was no sex difference for IL-6, pentraxin 3, IL-10, TGF-β1, TNF-α, IL-6Ra, glycoprotein 130 (gp130), TNF-α-R1, and TNF-α-R2 circulating levels (Supplementary Table 2A and B, Supplementary Methods).

**Table 1:** Characteristics of participants by sex (*n* = 1121)

	Women (*n* = 620)	Men (*n* = 501)	*p* value	*q* value
Age (years)	70.7 ± 3.9	71.0 ± 4.1	NS	NS
Weight (kg)	67.7 ± 11.2	80.6 ± 12.6	< 2.2*e*−16	< 2.2*e*−16
Height (cm)	160.0 ± 6.7	173.0 ± 6.4	< 2.2*e*−16	< 2.2*e*−16
BMI (kg/m^2^)	26.5 ± 4.1	26.9 ± 3.7	1.16*e*−02	NS
Hip circumference (cm)	103.3 ± 9.1	101.5 ± 7.6	1.32*e*−03	NS
Waist circumference (cm)	86.9 ± 10.8	96.7 ± 11.1	< 2.2*e*−16	< 2.2*e*−16
Waist-to-hip ratio	0.85 ± 0.31	0.95 ± 0.06	< 2.2*e*−16	< 2.2*e*−16
Calorie intake (kcal)	1680.9 ± 327.8	2123.3 ± 445.0	< 2.2*e*−16	< 2.2*e*−16
PASE score	127.8 ± 48.9	140.9 ± 59.5	3.53*e*−04	NS
Metabolic parameters				
Glucose	5.52 ± 0.77	5.85 ± 0.95	7.92*e*−11	1.54*e*−07
Insulin	8.75 ± 5.57	10.03 ± 7.85	NS	NS
HOMA IR	2.21 ± 1.58	2.70 ± 2.36	5.47*e*−03	NS
HOMA beta	90.43 ± 52.88	89.06 ± 63.57	2.08*e*−02	NS
Hemoglobin (g/dl)	13.7 ± 0.9	14.9 ± 1.0	< 2.2*e*−16	5.66*e*−14
Body composition markers				
FM (kg)	26.2 ± 8.06	22.0 ± 8.37	< 2.2*e*−16	< 2.2*e*−16
FMI (kg/m^2^)	10.3 ± 3.16	7.35 ± 2.74	< 2.2*e*−16	< 2.2*e*−16
LM (kg)	40.3 ± 4.97	57.1 ± 6.71	< 2.2*e*−16	< 2.2*e*−16
ALMI (kg/m^2^)	6.56 ± 0.77	8.47 ± 0.87	< 2.2*e*−16	< 2.2*e*−16
LMI (kg/m^2^)	15.7 ± 1.53	19.1 ± 1.80	< 2.2*e*−16	< 2.2*e*−16
FM/LM	0.65 ± 0.19	0.39 ± 0.14	< 2.2*e*−16	< 2.2*e*−16
SMI	0.25 ± 0.03	0.32 ± 0.04	< 2.2*e*−16	< 2.2*e*−16
BMC (g)	2092.5 ± 357	2947.8 ± 483	< 2.2*e*−16	< 2.2*e*−16
BMD (g/cm^2^)	1.03 ± 0.11	1.19 ± 0.11	< 2.2*e*−16	< 2.2*e*−16
T-score	− 0.82 ± 1.20	− 0.19 ± 1.20	< 2.2*e*−16	4.92*e*−14
Android/gynoid FM*	0.50 ± 0.15	0.78 ± 0.21	< 2.2*e*−16	< 2.2*e*−16
Android FM/LM*	0.79 ± 0.30	0.61 ± 0.25	2.70*e*−16	4.92*e*−13
Inflammatory parameters				
Ghrelin (pg/ml)	1631.46 [842.57–4427.87]	1256.32 [582.13–3538.03]	9.86*e*−05	
Leptin (ng/ml)	4.39 [2.86–6.21]	1.86 [0.94–3.16]	< 2.2*e*−16	< 2.2*e*−16
Adiponectin (μg/ml)	14.09 [9.76–19.96]	7.33 [5.03–10.51]	< 2.2*e*−16	< 2.2*e*−16
Resistin (pg/ml)	5850.83 [4287.64–7520.41]	6222.25 [4756.82–8310.03]	5.67*e*−03	
CRP (mg/l)	0.87 [0.44–1.72]	0.84 [0.41–1.78]	NS	NS
AGP (mg/ml)	0.67 [0.57–0.79]	0.61 [0.51–0.73]	1.24*e*−08	2.32*e*−05
Albumin (g/l)	44.90 [42.50–47.50]	44.95 [42.78–48.00]	NS	NS

*NS* not significant

* (Women, *n* = 474; Men, *n* = 416)

### Association of body composition with markers of inflammation and adipose-related hormones

**Fig. 1:**
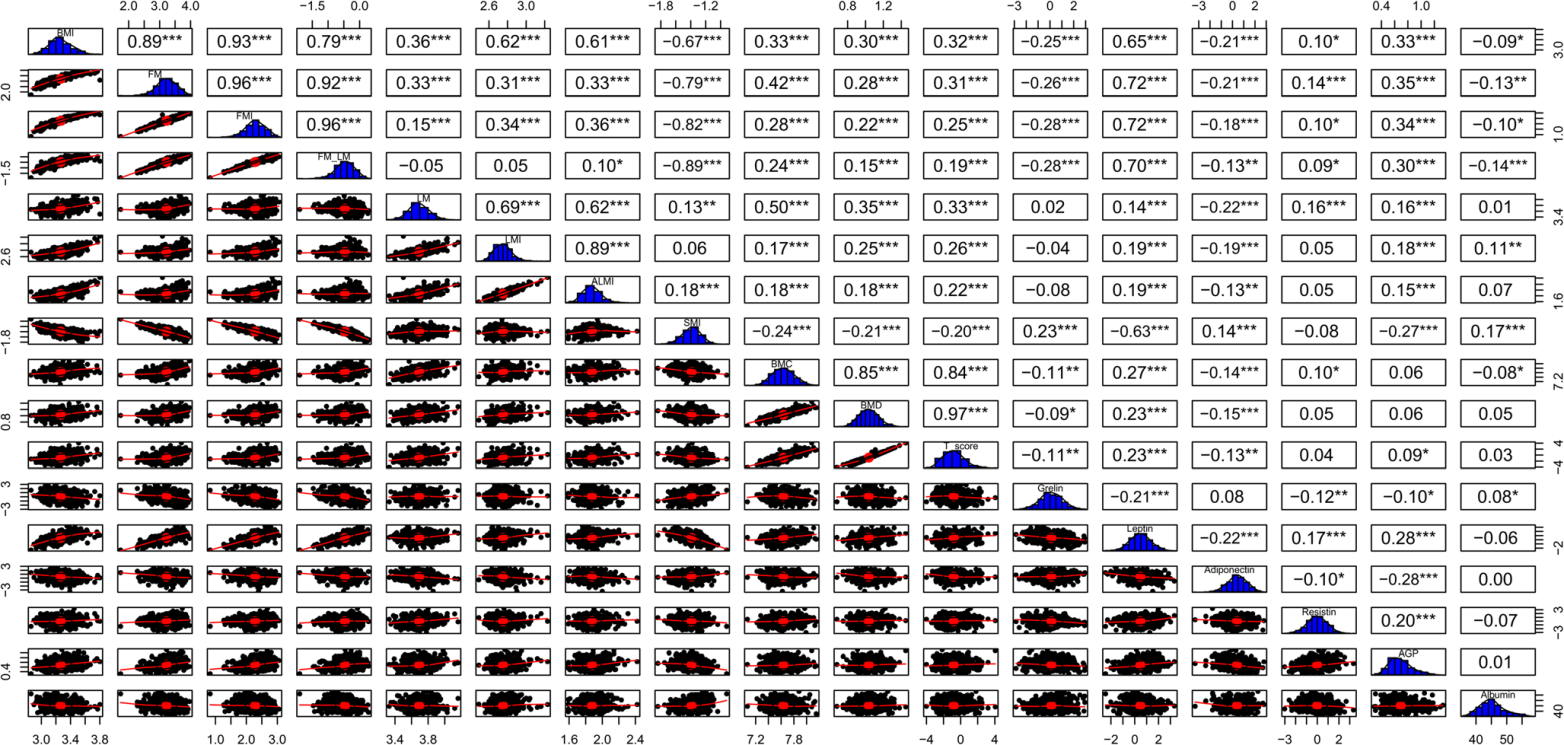
Pairwise scatter plot matrix. Histogram and correlation coefficients of all body composition parameters and inflammatory parameters in women. Pairwise scatter plots are in the lower triangle boxes, histograms are in the diagonal boxes, and correlation coefficients between variables are in the upper triangle boxes

**Fig. 2:**
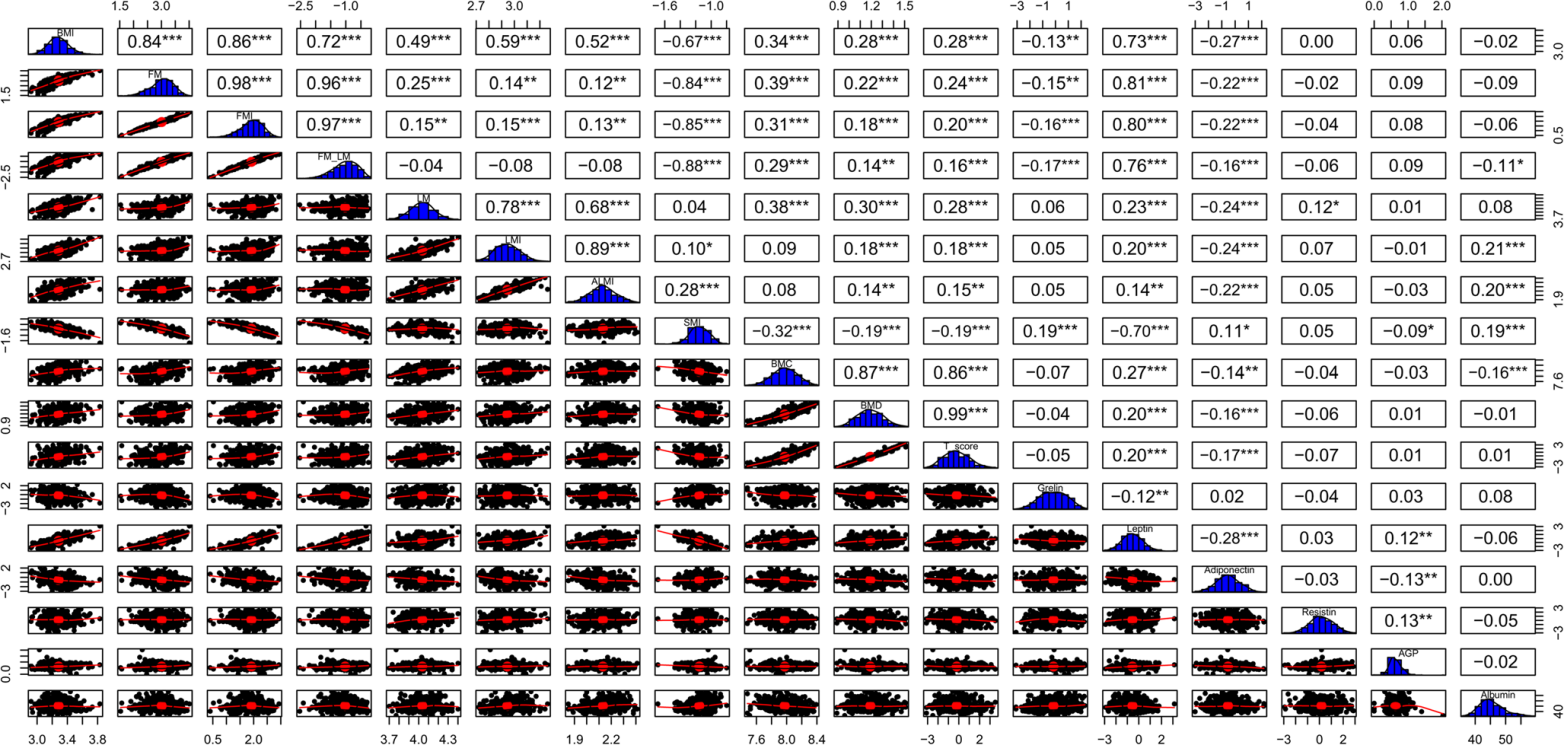
Pairwise scatter plot matrix. Histogram and correlation coefficients of all body composition parameters and inflammatory parameters in men. Pairwise scatter plots are in the lower triangle boxes, histograms are in the diagonal boxes and correlation coefficients between variables are in the upper triangle boxes

Significant associations of BC markers with inflammation and adipose-related hormones are summarized in Supplementary Table 3A and B. In elderly women, a negative correlation between ghrelin and adiposity was found, but not in men, where ghrelin showed a positive correlation with SMI. In both women and men, leptin showed strong positive associations with fat mass, while weak positive associations with lean mass and bone mass markers. A strong negative association was observed with SMI in both sexes. Resistin was not associated with any BC marker in both women and men, while no associations with BC markers were found in women with albumin and in men with AGP. Pairwise scatter plots reporting all the correlations between these markers in women and in men are shown in Figs. 1 and 2, respectively. As expected, there were significant correlations between all of the body composition markers; in particular, all the fat mass, lean mass, and bone markers are positively related, while the SMI is negatively correlated with the fat mass markers BMI, FM, FMI, and FM/LM in both female and male elderly subjects as reported in the upper left part of the pairwise scatter matrix plot in Figs. 1 and 2.

No significant correlation was found between the body composition markers and the following pro- and anti-inflammatory markers in both female and male elderly subjects: IL-6, pentraxin 3, IL-10, TGF-β1, TNF-α, IL-6Rα, gp130, TNF-α-R1, and TNF-α-R2 (Supplementary Table 2A and B).

Markers of inflammation and adiposity were also correlated with android FM/LM and android/gynoid FM (Tables 2 and 3). In women, positive correlations with android FM/LM and android/gynoid FM were found for leptin, CRP, and AGP; positive associations were only found for leptin and CRP in men. Android FM/LM and android/gynoid FM were negatively correlated with ghrelin, adiponectin, and albumin in women, while in men, a negative association was found with adiponectin.

**Table 2:** Correlation matrix for android fat mass/lean mass and android/gynoid fat mass with inflammatory and adiposity related markers in women

	ANDR FM/LM	ANDR/GYN FM	Ghrelin	Leptin	Adiponectin	Resistin	CRP	AGP	Albumin	IL-6	IL-6Rα	gp130	Pentraxin 3	TNF-α	TNF-α-R1	TNF-α-R2	IL-10	TGF-β1
ANDR FM/LM	*1*																	
ANDR/GYN FM	.76***	1																
Ghrelin	− .30***	− .20*	1															
Leptin	.68***	.42***	− .22***	1														
Adiponectin	− .27***	− .48***	.08	− .23***	1													
Resistin	.08	.04	− .09	.17*	− .10	1												
CRP	.28***	.22**	− .17	.24***	− .16	.12	1											
AGP	.32***	.31***	− .12	.27***	− .28***	.18*	.47***	1										
Albumin	− .27***	− .21**	.09	− .06	.00	− .08	− .08	.00	1									
IL-6	− .05	− .02	.30***	− .13	− .03	− .07	.03	.07	.01	1								
IL-6Rα	.00	.01	.10	.09	.02	.19	− .02	− .02	− .10	− .12	1							
gp130	.02	− .02	− .01	.08	.10	.11	− .05	− .11	− .13	− .14	.70***	1						
Pentraxin 3	.09	.00	− .20	.11	.14	− .02	.01	.00	− .11	− .09	.42***	.63***	1					
TNF-α	.04	.04	.28***	− .05	.00	− .12	.03	.02	.01	.63***	− .12	− .11	− .12	1				
TNF-α-R1	.19	.10	− .05	.24*	− .04	.25**	.08	.08	− .14	− .11	.65***	.78***	.52***	− .07	1			
TNF-α-R2	.20	.12	.01	.22	.01	.23*	.13	.07	− .19	− .05	.68***	.77***	.57***	− .04	.83***	1		
IL-10	− .03	− .01	.30***	− .11	.03	− .08	− .01	− .01	.02	.63***	− .09	− .07	− .13	.64***	− .04	.00>	1	
TGF-β1	.13	.09	− .08	− .04	− .04	.03	.05	.05	.07	.18**	− .40***	− .37***	− .23*	.19**	− .28***	− .31***	.15	1

*p < .05; **p < .01; ***p < .001

**Table 3:** Correlation matrix for android fat mass/lean mass and android/gynoid fat mass with inflammatory and adiposity-related markers in men

	ANDR FM/LM	ANDR/GYN FM	Ghrelin	Leptin	Adiponectin	Resistin	CRP	AGP	Albumin	IL-6	IL-6Rα	gp130	Pentraxin 3	TNF-α	TNF-α-R1	TNF-α-R2	IL-10	TGF-β1
ANDR FM/LM	1																	
ANDR/GYN FM	.79***	1																
Ghrelin	− .15	− .10	1															
Leptin	.76***	.56***	− .13	1														
Adiponectin	− .25***	− .43***	.01	− .27***	1													
Resistin	− .01	.05	− .03	.03	− .04	1												
CRP	.30***	.30***	− .06	.25***	− .16	.10	1											
AGP	.20	.22*	.04	.13	− .15	.12	.45***	1										
Albumin	− .19	− .12	.07	− .05	− .01	− .05	− .14	− .04	1									
IL-6	.08	.08	.35***	− .02	.02	.04	.22***	.22**	− .05	1								
IL-6Rα	− .01	− .06	.05	.03	.05	− .02	− .10	− .07	− .08	− .12	1							
gp130	− .11	− .15	− .03	− .07	.14	− .03	− .15	− .05	− .04	− .08	.72***	1						
Pentraxin 3	− .11	− .12	− .21	− .11	.19	− .07	− .08	.01	− .06	− .08	.57***	.70***	1					
TNF-α	− .02	.00	.41***	− .11	− .02	.01	.03	.08	.04	.64***	− .09	− .01	− .16	1				
TNF-α-R1	− .02	− .04	.03	.01	.00	.16	.02	.06	− .12	.00	.64***	.77***	.61***	.03	1			
TNF-α-R2	.03	− .01	.06	.07	.06	.19	.02	.08	− .16	.03	.66***	.76***	.63***	.01	.85***	1		
IL-10	.02	.03	.32***	− .09	.00	.03	.01	.10	− .08	.51***	.07	.09	− .04	.66***	.09	.12	1	
TGF-β1	.09	.08	− .11	− .01	.02	.12	.02	.07	− .02	.03	− .37***	− .32**	− .28*	− .03	− .28*	− .29*	.03	1

*p < .05; **p < .01; ***p < .001

### Association of markers of inflammation and adipose-related hormones with body composition clusters

Inflammatory markers and adipose-related hormones were also evaluated in relation to clusters of body composition markers that have been previously identified by the authors [22] (Supplementary Fig. 1).

**Fig. 3:**
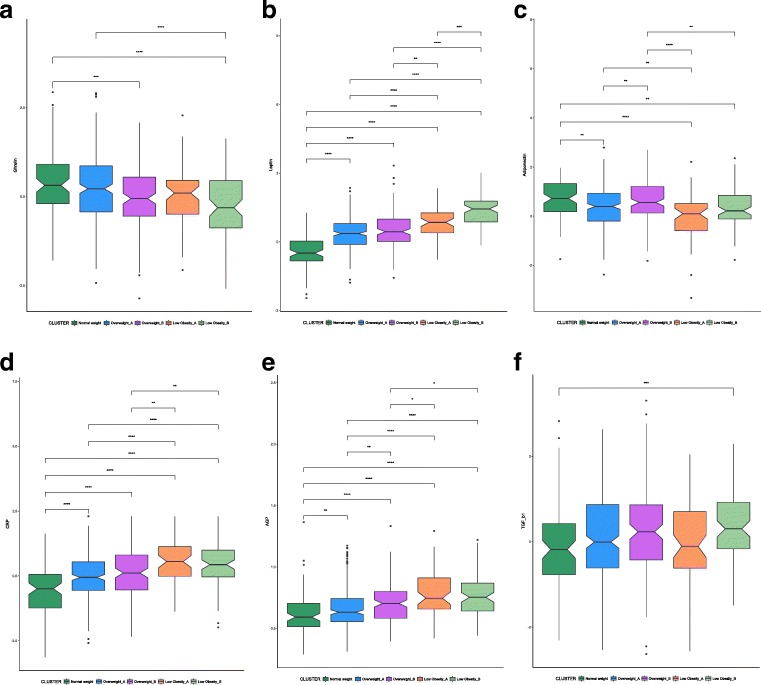
**a**–**f** Box plots and significant differences of inflammatory and adipose-related markers among clusters in women (*p* values: *p ≤ .05, **p ≤ .01, ***p ≤ .001)

Among the five clusters identified in women, there is a significant difference for ghrelin (*p* = 5.297*e*−06), adiponectin (*p* = 2.829*e*−06), CRP (*p* = 1.154*e*−12), leptin (*p* < 2.2*e*−16), AGP (*p* = 1.651*e*−12), and TGF-β1 (*p* = .005) (Fig. 3). Ghrelin levels are higher in the NW cluster compared with the OWB and LOB clusters, and the levels in the OWA are also higher than those in the LOB (Fig. 3a). Leptin levels are lower in the NW cluster of women compared with all the other four clusters, and interestingly, the LOB has significantly higher leptin levels than the LOA (Fig. 3b). Women belonging to the NW cluster have higher levels of adiponectin compared with OWA, LOA, and LOB (Fig. 3c). The levels of CRP are lower in the NW cluster compared with all the other four clusters (Fig. 3d). The levels of AGP are lower in the NW cluster compared with all the other four clusters (Fig. 3e). Among the five clusters identified in women, the levels of TGF-β1 are significantly different between NW and LOB (Fig. 3f).

**Fig. 4:**
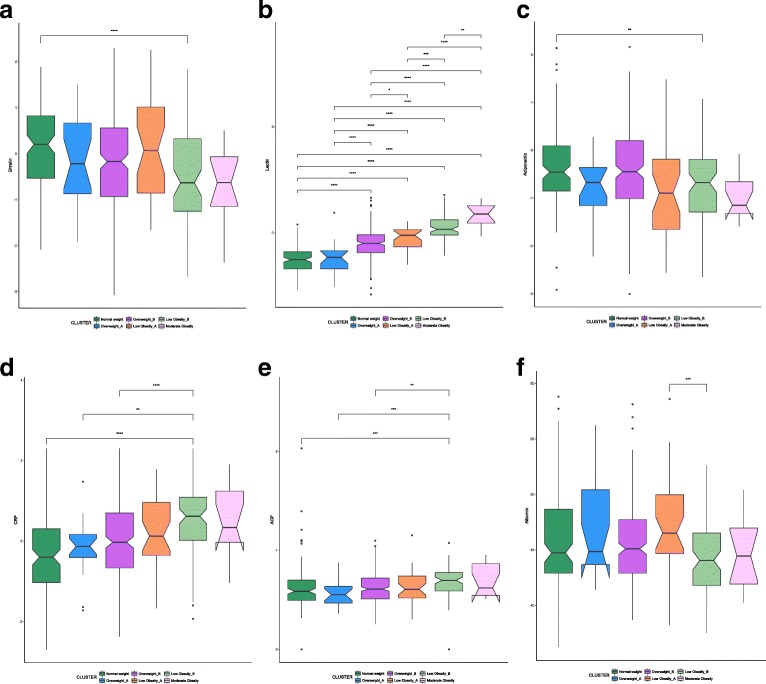
**a**–**f** Box plots and significant differences of inflammatory and adipose-related markers among clusters in men (*p* values: *p ≤ .05, **p ≤ .01, ***p ≤ .001)

Among the six clusters identified in men, there is a significant difference for ghrelin (*p* = .0006417), adiponectin (*p* = .0005453), CRP (*p* = 1.174*e*−06), leptin (*p* < 2.2*e*−16), albumin (*p* = .004843), and AGP (*p* = .001147) (Fig. 4). In particular, ghrelin levels are significantly higher in the NW cluster compared with the LOB (Fig. 4a). Leptin levels are lower in the NW cluster of men compared with the OWB, LOA, LOB, and MO (Fig. 4b). Adiponectin levels are higher in elderly men belonging to the NW cluster compared with the LOB cluster (Fig. 4c). CRP and AGP levels are significantly lower in elderly men compared with the NW and LOB clusters, OWA and LOB clusters, and OWB and LOB clusters (Fig. 4d, e). Albumin is significantly higher in the LOA compared with the LOB elderly men (Fig. 4f).

## Discussion

The present study reports evidence for an association between body composition markers assessed by DXA and the concentration of a number of pro- and anti-inflammatory parameters as well as adipose-related hormones. Although DXA does not provide a direct measurement of FM, LM, and BMC, it is widely used for BC assessment in both clinical and research settings, because of its good values of accuracy and precision, large availability, low costs, low radiation dose, and good correlations with BC measurements obtained by CT and MRI [21, 23–25].

Although several studies have shown specific associations with central and/or peripheral fat mass, BMI and waist circumference, and inflammatory molecules such as IL-1Rα, IL-6, and IL-6-sR [26–28], we did not find any correlation with the body composition markers and indexes studied and circulating levels of a series of pro- and anti-inflammatory molecules such as IL-6, pentraxin 3, IL-10, TGF-β1, TNF-α, IL-6Rα, gp130, TNF-α-R1, and TNF-α-R2. This difference could be explained by the size of the cohort used, by the technique used to identify body composition and many other factors.

As expected, major differences exist between BC characteristics in elderly women and men. Elderly women have higher fat mass than men while men have higher lean mass and bone content than elderly women. Sex dimorphism in total body composition is present at birth and continues through adulthood [29]. Men maintain their lean mass into the fifth decade of life but then begin to lose muscle mass due to both hormonal changes, decline in activity levels, low protein diet, reduced blood flow, and decreased nerve conduction. Women show a similar decline in lean mass, but they often show greater gains in fatness [29], even when weight is stable [30]. Such changes continue into old age [4, 31].

Among the adipose-related markers, ghrelin, which is considered an anti-inflammatory molecule, is negatively associated with fat mass in women but not in men, while it is positively associated with SMI in both sexes. Ghrelin levels are reported to fall in obesity, with concentration influenced principally by changes in energy balance. Insulin, in particular, may play an important role in the decrease of ghrelin levels after meals [32]. Even if BMI and insulin are not different between sexes, women have greater fat mass than men, and this could explain the different associations found. Also when comparing the ghrelin levels among the five clusters previously identified by the authors [22] which differ for BMI and fat mass and lean mass and bone density, a sex difference emerged. In women, the levels of ghrelin decrease as BMI clusters increase from normal weight to low obesity clusters.

In both men and women, leptin is positively associated with fat mass, lean mass, and bone mass markers, while it is negatively associated with SMI. Leptin is a classic adipokine that is secreted by adipocytes, and it increases with weight gain and decreases with weight loss and is also considered as a pro-inflammatory marker [33]. Recent studies have reported, however, that leptin is also produced by skeletal muscle [34, 35] as well as bone cells [36]. Leptin treatment increases muscle mass and decreases the expression of atrophy-related factors such as myostatin, muscle RING-finger protein-1 (MuRF1), and muscle atrophy F-box (MAFbx) in muscle [37] without any change with age. More recent studies show that the effects of leptin on the skeleton are quite complex, and that leptin deficiency is associated with low bone mass primarily due to reduced cortical bone [38, 39]. Central infusions of leptin in leptin-deficient ob/ob mice actually increase cortical bone formation and total bone mass [40]. Individuals with osteoporosis have reduced levels of leptin in the bone marrow microenvironment [41].

Adiponectin, together with leptin, is able to regulate energy homeostasis. Low levels of adiponectin, that is considered an anti-inflammatory mediator, have been found in obesity and insulin resistance [15]. In our cohort, both men and women show an inverse relationship with fat and lean mass markers. Our results are in agreement with a recent paper by Baker and colleagues [42] showing that in elderly, high levels of serum adiponectin are correlated with low BMI, fat, and lean mass BC markers. Moreover, adiponectin levels decrease as clusters increase BMI in both sexes. However, it is interesting to note that in women when comparing clusters with similar BMI (25.09 and 26.62, respectively), adiponectin levels are higher in the overweight group with higher levels of fat and bone mass and lower levels of lean mass. In men, the levels of adiponectin are generally not different among the six clusters.

It has been reported that an increase in fat mass is correlated with markers of inflammation among community-dwelling individuals older than 65 years [26, 27]. The mechanisms inducing obesity-related inflammation are not completely understood; however, the expansion of adipose tissue in response to a positive energy balance may play a major role. When adipose tissue expands, it leads to the activation of macrophages which secrete inflammatory cytokines including TNF-α and IL-6 [43]. In addition, leptin together with resistin could also function as a pro-inflammatory molecule in the presence of obesity [44], while adiponectin and ghrelin have anti-inflammatory properties. In particular, adiponectin is known to inhibit inflammation by blocking NF-kB activation and reducing such cytokines as TNF-α, IL-6, and IL-18 [45, 46]. Moreover, adiponectin may also play a pro-inflammatory role in arthritic joints by promoting COX2 expression and the synthesis of PGE_2_, which increases inflammation and pain [47]. Through the elaboration of anti- and pro-inflammatory adipokines that enter the systemic circulation, adipose tissue plays a critical role in regulating the inflammatory response in the setting of calorie restriction, obesity, and aging. However, it is possible that the association with inflammatory markers differs by sex and by adipose tissue location. The most commonly measured inflammatory proteins in nutrition surveys are CRP, which is a measure of acute inflammation, and AGP, which is a measure of chronic inflammation [48]. In our study, CRP correlated with fat mass in both sexes, but only in women with LMI and ALMI. CRP and AGP levels gradually increased as the BMI increased in the clusters in women. It has been shown that the effects of aging on the human immune system are significantly different in men and women, showing a stronger pro-inflammatory response in women [49]. Even if in our study any difference emerged in CRP concentrations between men and women (median 0.84 mg/l and 0.87 mg/l, respectively), women have a significantly higher concentration of AGP compared to men (median 0.67 g/l and 0.61 g//l, respectively). Indeed, AGP was positively correlated with fat markers and LMI only in women. Hemoglobin levels were also significantly lower in women than in men (median 13.7 g/dl and 14.9 g/dl, respectively), and these lower levels of iron could possibly further contribute to the different inflammatory status [48] between men and women. CRP acts as a positive and albumin as a negative acute-phase reactant [50]. This seems to provide a link to the already mentioned, slightly increased inflammatory state in elderly women. In this context, it is interesting that several clinical studies could demonstrate a link between the specific pattern of increased CRP and decreased albumin concentrations with sarcopenia, frailty, and vascular and non-vascular mortality in elderly subjects [50–52].

Among the body composition markers, SMI associates differently from the others with the adipose-related and inflammatory markers analyzed in this study. SMI represents a marker of sarcopenia, together with ALMI [53, 54]. In our cohort, the associations of SMI with adipose-related and inflammatory markers studied are always discordant in both women and men, the only exception being the positive correlation with albumin levels in men. In particular, in both women and men, SMI correlated positively with ghrelin, which is considered an anti-inflammatory molecule, but negatively with leptin, CRP, and AGP, which are considered pro-inflammatory markers. As inflammation is thought to have a role in age-related sarcopenia [55], the results obtained with SMI are more consistent with respect to those obtained with ALMI when both are considered as markers of sarcopenia. These results fit with the open debate on the use of optimal quantitative markers of sarcopenia and the role of imaging [53, 56]. Moreover, SMI is inversely correlated with BMI and fat mass markers and positively with ALMI but not with LM and LMI, while ALMI is positively correlated with BMI and fat mass markers and also with LM and LMI. These results showed that it is likely that ALMI still represents the general lean mass instead of being a marker of sarcopenia; however, further studies are needed to verify this hypothesis. These last results could be of help in the prevention of sarcopenia.

## Electronic supplementary material

ESM 1 (DOCX 74.1 kb)

## Abbreviations

AGP: Alpha-1-acid glycoprotein
ALMI: Appendicular lean mass index
BC: Body composition
BMC: Bone mineral content
BMD: Bone mineral density
CVD: Cardiovascular disease
DXA: Dual-energy X-ray absorptiometry
FM: Fat mass
FMI: Fat mass index
gp130: Glycoprotein 130
hsCRP: High-sensitivity C-reactive protein
IL: Interleukin
IL-6Rα: Interleukin-6 receptor alpha
LOA: Low obesity A
LOB: Low obesity B
LM: Lean mass
LMI: Lean mass index
MO: Moderate obesity
NW: Normal weight
OWA: Overweight A
OWB: Overweight B
SMI: Skeletal muscle mass index
TGF-β1: Transforming growth factor beta 1
TNF-α: Tumor necrosis factor alpha
TNF-α-R: TNF-α receptor
