# Supplementary material for: Gender-specific association of body composition with inflammatory and adipose-related markers in healthy elderly Europeans from the NU-AGE study
Source: Eur Radiol. 2019 Feb 4;29(9):4968–79. doi: 10.1007/s00330-018-5973-2 (PMC6682581; doi:10.1007/s00330-018-5973-2)
Supplement: Supplementary file 1 — (DOCX 74.1 kb) [file 330_2018_5973_MOESM1_ESM.docx]

**SUPPLEMENTARY METHODS**

**Assessment of Body Composition**

Lunar iDXA, GE Healthcare– enCORETM 2011 software version 13.6 (Bologna, Italy); Discovery QDR, Hologic Inc.– software version 3 (Clermont-Ferrand, France); Lunar Prodigy, GE Healthcare– enCORETM 2011 software version 13.6 (Wageningen, the Netherlands and Warsaw, Poland); and Discovery Wi, Hologic Inc., (Norwich, UK). The scanners followed standard Quality Control procedures and they were calibrated daily using a standard calibration block supplied by the manufacturers. DXA scans were performed by trained technicians according to state-of-the-art technique and manufacturers recommendation.

All metal items were removed before densitometry. Participants were placed in a supine position with arms at sides slightly separated from the trunk and correctly centered on the scanning field.

**Markers of inflammation and adipose related hormones**

Each recruiting center collected fresh blood samples from each participant after fasting. Blood was immediately centrifuged at 2000 x g for 10 min at 4°C and separated into plasma and serum according to a standardized operating procedure. All the specimens were stored at -80 °C until the time of analysis and sent to the project partners responsible for the analyses of the markers of inflammation and adipose-related hormones. Inflammatory and adipose-related markers were analyzed by a magnetic bead-based multiplex immunoassays (Bio-Plex) (Bio-Rad) according to the manufacturer’s instructions. In particular Interleukin 6 (IL-6), Interleukin 10 (IL-10), and Tumor Necrosis Factor alpha (TNFα) were measured in multiplex with Bio-Plex Pro Cytokine, Chemokine, and Growth Factor Assays (IL-6 intra-assay coefficient of variation (CV), 4.01%; IL-10 intra-assay CV, 3.99%; TNFα intra-assay CV, 4.55%); Transforming Growth Factor beta1 (TGF-β1 intra-assay CV, 3.83%) with Bio-Plex Pro TGF-beta assay; Ghrelin (inter-assay CV, 2%) and Resistin (inter-assay CV, 4%) in multiplex with Bio-Plex Pro human diabetes assay. Plates were read and analyzed by Bio-Plex Manager Software. The level of Interleukin 6 receptor alpha (IL-6Rα, inter-assay CV, 3.1%)), Glycoprotein 130 (gp130, inter-assay CV, 5.9%), Pentraxin-3 (inter-assay CV, 6.8%) and soluble TNF alpha receptors R1 (TNFα-R1, inter-assay CV, 6.1%) and R2 (TNFα-R2, inter-assay CV, 7.7%) were assessed in multiplex in a subgroup of 569 samples with Bioplex Pro human inflammation assay (gp-130, inter-assay CV 5.9%).

The quantitative determination of high sensitivity C-reactive protein (hsCRP), leptin, adiponectin has been performed by ProcartaPlex^TM^ Immunoassay (Thermo Fisher Scientific) according to the manufacturer’s instructions. Analysis was performed using Luminex 200 instrumentation (Luminex Corporation). Assay sensitivities were 19.31 pg/mL for Leptin, 4.39 pg/mL for hsCRP, and 47.46 pg/mL for adiponectin.

α1 Acid glycoprotein (AGP) has been measured by an immunoturbidimetric assay (AAGP2, Tina-quant α1-Acid Glycoprotein Gen.2 COBAS, Roche Diagnostics) with a measuring range of 0.1-4.0 g/L. Plasma albumin level was analyzed using the VITROS ALB slides (Ortho-Clinical Diagnostics) on a Vitros 5.1/FS analyzer. Method CV: 0.9 -1.7%

**SUPPLEMENTARY MATERIALS**

**Supplementary Table 1:**

| Clusters | BMI  (kg/m^2^) | FM  (kg) | FMI  (kg/m^2^) | LM  (kg) | LMI  (kg/m^2^) | ALMI  (kg/m^2^) | FM/LM | SMI | T-score | BMC  (g) | BMD  (g/cm^2^) |
| --- | --- | --- | --- | --- | --- | --- | --- | --- | --- | --- | --- |
| Normal weight  (NW; n = 89; 14.4%) | 21.4±1.7 | 15.9±3.4 | 6.1±1.3 | 38.4±3.1 | 14.9±1.1 | 6.2±0.6 | 0.4±0.1 | 0.29±0.03 | -1.4±1.0 | 1905.1±230.9 | 1.0±0.1 |
| Overweight A  (OWA; n = 251; 40.5%) | 25.1±1.9 | 23.8±4.0^1^ | 9.2±1.5^1^ | 40.4±4.1^1^ | 15.5±1.1^1^ | 6.4±0.5 | 0.6±0.1^1^ | 0.26±0.02^1^ | -0.4±1.0^1^ | 2190.4±294.9^1^ | 1.1±0.1^1^ |
| Overweight B  (OWB; n = 137; 22.1%) | 26.6±2.7 | 26.9±5.9 | 10.9±2.3 | 37.2±3.7 | 15.1±1.0 | 6.3±0.6 | 0.7±0.2 | 0.24±0.02 | -1.9±0.8 | 1804.1±249.5 | 0.9±0.1 |
| Low Obesity A  (LOA; n = 61; 9.8%) | 31.5±4.1 | 32.7 ±6.4^a^ | 12.9 ±2.6^a^ | 47.1±5.8 ^a^ | 18.6 ±1.7^a^ | 8.0±0.8 ^a^ | 0.7±0.1^a^ | 0.25±0.02 ^a^ | -0.6±1.5^a^ | 2133.4±378.2^a^ | 1.1±0.1 ^a^ |
| Low Obesity B  (LOB; n = 82; 13.2%) | 31.9±2.4 | 38.5±5.4 | 14.9±2.0 | 42.1±4.4 | 16.2±1.1 | 6.8±0.6 | 0.9±0.1 | 0.21±0.02 | 0.2±0.8 | 2454.4±297.3 | 1.1±0.1 |

**A: Five body composition groups identified by a cluster analysis performed on ten BC markers and BMI in elderly women participants (N=620)**

Values are expressed as mean values ± SD, unless otherwise indicated.

^1,^Significant difference between OWA and OWB (^1^: p<0.0001)

^a,^Significant difference between LOA and LOB (^a^: p<0.0001)

**B: Six body composition groups identified by a cluster analysis performed on ten BC markers and BMI in elderly men participants (N=501)**

| Clusters | BMI  (kg/m^2^) | FM  (kg) | FMI  (kg/m^2^) | LM  (kg) | LMI  (kg/m^2^) | ALMI  (kg/m^2^) | FM/LM | SMI | T-score | BMC  (g) | BMD  (g/cm^2^) |
| --- | --- | --- | --- | --- | --- | --- | --- | --- | --- | --- | --- |
| Normal weight  (NW; n = 122; 24.4%) | 24.0±2.1 | 13.7±4.2 | 4.6±1.4 | 57.0±5.5 | 19.2±1.4 | 8.5±0.7 | 0.2±0.1 | 0.36±0.03 | -0.9±1.0 | 2631.9±418.8 | 1.1±0.1 |
| Overweight A  (OWA; n = 20; 4.0%) | 25.7±2.8 | 15.6±5.3^1^ | 5.1±1.6^1^ | 61.8±6.6^1^ | 20.3±1.3^1^ | 9.1±0.6^1^ | 0.2±0.1^1^ | 0.36±0.03^1^ | 1.9±0.6^1^ | 3576.2±401.1^1^ | 1.4±0.1^1^ |
| Overweight B  (OWB; n = 233; 46.5%) | 26.3±2.3 | 22.2±5.3 | 7.5±1.8 | 54.0±5.3 | 18.2±1.3 | 8.1±0.7 | 0.4±0.1 | 0.31±0.02 | -0.4±0.9 | 2891.4±331.9 | 1.2±0.1 |
| Low Obesity A  (LOA; n = 34; 6.8%) | 30.1±1.6 | 23.3±4.8^a^ | 7.8 ±1.5^a^ | 65.8±5.2 ^a^ | 22.0±1.1 ^a^ | 9.8±0.6 ^a^ | 0.4±0.1 ^a^ | 0.32±0.03 ^a^ | -0.5±0.9 ^a^ | 2791.9±347.2 ^a^ | 1.6±0.1 ^a^ |
| Low Obesity B  (LOB; n = 80; 16.0%) | 30.4±2.9 | 31.5±5.4 | 10.3±1.9 | 59.5±5.8 | 19.3±1.6 | 8.5±0.8 | 0.5±0.1 | 0.28±0.02 | 0.7±1.1 | 3391.6±432.8 | 1.3±0.1 |
| Moderate Obesity  (MO; n = 12; 2,3%) | 36.6±2.9 | 42.4±5.1 | 13.9±1.5 | 67.3±7.8 | 21.9±2.0 | 8.5±1.1 | 0.6±0.1 | 0.26±0.03 | 1.6±1.2 | 3667.6±637.3 | 1.3±0.1 |

Values are expressed as mean values ± SD, unless otherwise indicated.

^1,^Significant difference between OWA and OWB (^1^: p<0.0001)

^a,^Significant difference between LOA and LOB (^a^: p<0.0001)

**Supplementary Table 2A: Correlation Matrix for Body Composition Markers with pro- and anti-inflammatory markers in women.** *p < .05. **p < .01. ***p < .001

|  | BMI | FM | FMI | FM/LM | LM | LMI | ALMI | SMI | BMC | BMD | T-score | IL-6 | IL-6Rα | gp130 | Pentraxin-3 | TNFα | TNFα-R1 | TNFα-R2 | IL -10 | TGF-β1 |
| --- | --- | --- | --- | --- | --- | --- | --- | --- | --- | --- | --- | --- | --- | --- | --- | --- | --- | --- | --- | --- |
| BMI | 1 |  |  |  |  |  |  |  |  |  |  |  |  |  |  |  |  |  |  |  |
| FM | .90^***^ | 1 |  |  |  |  |  |  |  |  |  |  |  |  |  |  |  |  |  |  |
| FMI | .93^***^ | .96^***^ | 1 |  |  |  |  |  |  |  |  |  |  |  |  |  |  |  |  |  |
| FM/LM | .80^***^ | .92^***^ | .96^***^ | 1 |  |  |  |  |  |  |  |  |  |  |  |  |  |  |  |  |
| LM | .38^***^ | .34^***^ | .16 | -.05 | 1 |  |  |  |  |  |  |  |  |  |  |  |  |  |  |  |
| LMI | .61^***^ | .32^***^ | .35^***^ | .06 | .69^***^ | 1 |  |  |  |  |  |  |  |  |  |  |  |  |  |  |
| ALMI | .59^***^ | .34^***^ | .36^***^ | .11 | .63^***^ | .89^***^ | 1 |  |  |  |  |  |  |  |  |  |  |  |  |  |
| SMI | -.69^***^ | -.79^***^ | -.82^***^ | -.89^***^ | .12 | .05 | .17^*^ | 1 |  |  |  |  |  |  |  |  |  |  |  |  |
| BMC | .34^***^ | .43^***^ | .29^***^ | .25^***^ | .49^***^ | .17^*^ | .19^**^ | -.24^***^ | 1 |  |  |  |  |  |  |  |  |  |  |  |
| BMD | .29^***^ | .28^***^ | .22^***^ | .16 | .34^***^ | .23^***^ | .18^*^ | -.20^**^ | .85^***^ | 1 |  |  |  |  |  |  |  |  |  |  |
| T-score | .31^***^ | .31^***^ | .26^***^ | .19^**^ | .32^***^ | .24^***^ | .22^***^ | -.19^**^ | .84^***^ | .96^***^ | 1 |  |  |  |  |  |  |  |  |  |
| IL-6 | -.06 | -.05 | -.06 | -.05 | -.02 | -.05 | -.04 | .03 | -.05 | -.09 | -.06 | 1 |  |  |  |  |  |  |  |  |
| IL-6Rα | .00 | .00 | -.01 | -.02 | .04 | .02 | -.01 | -.01 | .06 | .06 | .04 | -.12 | 1 |  |  |  |  |  |  |  |
| gp130 | -.01 | -.01 | -.02 | -.02 | .02 | -.02 | -.04 | -.02 | .09 | .09 | .07 | -.14 | .70^***^ | 1 |  |  |  |  |  |  |
| Pentraxin-3 | .06 | .06 | .07 | .09 | -.06 | -.03 | .00 | -.08 | .03 | .04 | .04 | -.09 | .42^***^ | .63^***^ | 1 |  |  |  |  |  |
| TNFα | .03 | .02 | .03 | .03 | -.03 | .00 | -.02 | -.06 | -.01 | -.03 | .00 | .63^***^ | -.12 | -.11 | -.12 | 1 |  |  |  |  |
| TNFα-R1 | .16 | .19 | .16 | .16 | .11 | .06 | .05 | -.16 | .21 | .20 | .19 | -.11 | .65^***^ | .78^***^ | .52^***^ | -.07 | 1 |  |  |  |
| TNFα-R2 | .16 | .17 | .16 | .16 | .04 | .04 | .04 | -.16 | .13 | .11 | .09 | -.05 | .68^***^ | .77^***^ | .57^***^ | -.04 | .83^***^ | 1 |  |  |
| IL-10 | -.02 | -.03 | -.03 | -.03 | .01 | .00 | -.01 | .01 | -.02 | -.04 | -.04 | .63^***^ | -.09 | -.07 | -.13 | .64^***^ | -.04 | .00 | 1 |  |
| TGF-β1 | .09 | .09 | .09 | .11 | -.04 | -.02 | -.04 | -.14 | .07 | .06 | .04 | .18 | -.40^***^ | -.37^***^ | -.23^*^ | .19^*^ | -.28^***^ | -.31^***^ | .15 | 1 |

**Supplementary Table 2B: Correlation Matrix for Body Composition Markers with pro- and anti-inflammatory markers in men.** *p < .05. **p < .01. ***p < .001

|  | **BMI** | **FM** | **FMI** | **FM/LM** | **LM** | **LMI** | **ALMI** | **SMI** | **BMC** | **BMD** | **T-score** | **IL-6** | **IL-6Rα** | **gp130** | **Pentraxin-3** | **TNFα** | **TNFα-R1** | **TNFα-R2** | **IL-10** | **TGFβ1** |
| --- | --- | --- | --- | --- | --- | --- | --- | --- | --- | --- | --- | --- | --- | --- | --- | --- | --- | --- | --- | --- |
| BMI | 1 |  |  |  |  |  |  |  |  |  |  |  |  |  |  |  |  |  |  |  |
| FM | .84^***^ | 1 |  |  |  |  |  |  |  |  |  |  |  |  |  |  |  |  |  |  |
| FMI | .86^***^ | .98^***^ | 1 |  |  |  |  |  |  |  |  |  |  |  |  |  |  |  |  |  |
| FM/LM | .72^***^ | .96^***^ | .97^***^ | 1 |  |  |  |  |  |  |  |  |  |  |  |  |  |  |  |  |
| LM | .48^***^ | .25^***^ | .14 | -.04 | 1 |  |  |  |  |  |  |  |  |  |  |  |  |  |  |  |
| LMI | .59^***^ | .14 | .15 | -.08 | .78^***^ | 1 |  |  |  |  |  |  |  |  |  |  |  |  |  |  |
| ALMI | .51^***^ | .12 | .13 | -.08 | .68^***^ | .88^***^ | 1 |  |  |  |  |  |  |  |  |  |  |  |  |  |
| SMI | -.68^***^ | -.84^***^ | -.85^***^ | -.88^***^ | .04 | .10 | .29^***^ | 1 |  |  |  |  |  |  |  |  |  |  |  |  |
| BMC | .35^***^ | .39^***^ | .31^***^ | .29^***^ | .38^***^ | .10 | .08 | -.33^***^ | 1 |  |  |  |  |  |  |  |  |  |  |  |
| BMD | .28^***^ | .23^***^ | .19^*^ | .15 | .29^***^ | .18 | .13 | -.20^**^ | .88^***^ | 1 |  |  |  |  |  |  |  |  |  |  |
| T-score | .29^***^ | .24^***^ | .21^**^ | .17 | .28^***^ | .18 | .15 | -.19^**^ | .86^***^ | .98^***^ | 1 |  |  |  |  |  |  |  |  |  |
| IL-6 | .06 | .06 | .07 | .07 | -.03 | .01 | .01 | -.05 | -.11 | -.13 | -.11 | 1 |  |  |  |  |  |  |  |  |
| IL-6Rα | .09 | .04 | .05 | .03 | .04 | .09 | .15 | .02 | .06 | .03 | .04 | -.12 | 1 |  |  |  |  |  |  |  |
| gp130 | -.04 | -.06 | -.07 | -.07 | .05 | .02 | .03 | .08 | .10 | .08 | .09 | -.08 | .72^***^ | 1 |  |  |  |  |  |  |
| Pentraxin-3 | -.11 | -.10 | -.09 | -.08 | -.12 | -.08 | -.07 | .06 | -.01 | .01 | .02 | -.08 | .57^***^ | .70^***^ | 1 |  |  |  |  |  |
| TNFα | -.03 | -.03 | -.03 | -.03 | -.01 | .02 | .04 | .06 | -.09 | -.11 | -.10 | .64^***^ | -.09 | -.01 | -.16 | 1 |  |  |  |  |
| TNFα-R1 | .03 | .00 | .00 | -.03 | .08 | .09 | .09 | .05 | .06 | .05 | .06 | .00 | .64^***^ | .77^***^ | .61^***^ | .03 | 1 |  |  |  |
| TNFα-R2 | .08 | .08 | .07 | .06 | .09 | .07 | .08 | -.02 | .08 | .03 | .04 | .03 | .66^***^ | .76^***^ | .63^***^ | .01 | .85^***^ | 1 |  |  |
| IL-10 | .00 | -.04 | -.02 | -.03 | -.05 | .02 | .02 | .02 | -.10 | -.12 | -.12 | .51^***^ | .07 | .09 | -.04 | .66^***^ | .09 | .12 | 1 |  |
| TGF-β1 | .06 | .06 | .07 | .06 | .01 | .02 | -.04 | -.10 | -.04 | -.04 | -.06 | .03 | -.37^***^ | -.32^**^ | -.28^*^ | -.03 | -.28^*^ | -.29^*^ | .03 | 1 |

**Supplementary Table 3A:** Correlation Matrix of all Body Composition parameters and Inflammatory markers and adipose-related hormones in women *p < .05. **p < .01. ***p < .001

|  | BMI | FM | FMI | FM/LM | LM | LMI | ALMI | SMI | BMC | BMD | T Score |
| --- | --- | --- | --- | --- | --- | --- | --- | --- | --- | --- | --- |
| Ghrelin | -.26^***^ | -.27^***^ | -.29^***^ | -.30^***^ | NS | NS | NS | NS | NS | NS | NS |
| Leptin | .65^***^ | .71^***^ | .71^***^ | .70^***^ | NS | .19^**^ | .19^**^ | -.62^***^ | .27^***^ | .23^***^ | .22*** |
| Adiponectin | -.20^**^ | -.20^**^ | NS | NS | -.23^***^ | -.19^**^ | NS | NS | NS | NS | NS |
| CRP | .35^***^ | .36^***^ | .37^***^ | .33^***^ | NS | .19^**^ | .22^***^ | -.23^***^ | NS | NS | NS |
| AGP | .31^***^ | .33^***^ | .32^***^ | .28^***^ | NS | .17^*^ | NS | -.26^***^ | NS | NS | NS |

**Supplementary Table 3B:** Correlation Matrix of all Body Composition parameters and Inflammatory markers and adipose-related hormones in men *p < .05. **p < .01. ***p < .001

|  | BMI | FM | FMI | FM/LM | LM | LMI | ALMI | SMI | BMC | BMD | T Score |
| --- | --- | --- | --- | --- | --- | --- | --- | --- | --- | --- | --- |
| Ghrelin | NS | NS | NS | NS | NS | NS | NS | .19^*^ | NS | NS | NS |
| Leptin | .74^***^ | .81^***^ | .80^***^ | .76^***^ | .23^***^ | .19^*^ | NS | -.70^***^ | .27^***^ | .20^**^ | .20* |
| Adiponectin | -.26^***^ | -.22^**^ | -.21^**^ | NS | -.24^***^ | -.24^***^ | -.23^***^ | NS | NS | NS | NS |
| CRP | .23^***^ | .29^***^ | .29^***^ | .28^***^ | NS | NS | NS | -.24^***^ | NS | NS | NS |
| Albumin | NS | NS | NS | NS | NS | .20^*^ | .20^*^ | .20^*^ | NS | NS | NS |
